# Synthesis and crystal structures of (*E*)-*N*′-(4-chloro-3-nitro­benzyl­idene)acetohydrazide and (*E*)-2-(4-chloro­benzyl­idene)-1-(quinolin-8-yl)hydrazine

**DOI:** 10.1107/S2056989023006412

**Published:** 2023-07-28

**Authors:** Tamer Nasr, Benson M. Kariuki, Mai M. Elansary, Radwan Elhaggar, Wafaa Zaghary

**Affiliations:** a Department of Pharmaceutical Chemistry, Faculty of Pharmacy, Helwan University, 11795 Helwan, Cairo, Egypt; b Pharmaceutical Chemistry Department, Faculty of Pharmacy, Modern University for Technology and Information, MTI, Cairo, Egypt; c School of Chemistry, Cardiff University, Main Building, Park Place, Cardiff, CF10 3AT, United Kingdom; Universidad Nacional Autónoma de México, México

**Keywords:** synthesis, crystal structure, benzyl­idene, hydrazine

## Abstract

The syntheses of two benzyl­idenehydrazine derivatives, namely, (*E*)-*N*′-(4-chloro-3-nitro­benzyl­idene)acetohydrazide, C_9_H_8_ClN_3_O_3_, and (*E*)-2-(4-chloro­benzyl­idene)-1-(quinolin-8-yl)hydrazine, C_16_H_12_ClN_3_, are reported. The mol­ecules have been characterized using IR, ^1^H NMR, ^13^C NMR and mass spectro­scopic and elemental analysis techniques, and their structures have been determined by single-crystal X-ray diffraction.

## Abstract 2

Two benzyl­idenehydrazinederivatives have been synthesized and structurally characterized.

## 1. Chemical context

Quinolines are a major component of many natural products (Grundon, 1984 ▸) and drugs (Alhaider *et al.*, 1985 ▸; Campbell *et al.*, 1988 ▸). Compounds containing the quinoline ring system demonstrate a variety of biological and pharmaceutical activities (Marella *et al.*, 2013 ▸). In the pharmaceutical industry, medications with a quinoline ring are known to have a wide range of therapeutic uses. Commercially available drugs include anti­asthmatic (Montelukast) (Paggiaro & Bacci, 2011 ▸), anti­cancer (Irinotecan) (Ahmed *et al.*, 2022 ▸; Ammar *et al.*, 2021 ▸; Mandewale *et al.*, 2017 ▸), anti­viral (Saquinavir) (Kaur & Kumar, 2021 ▸), anti­bacterial (Ciprofloxacin) (Ezelarab *et al.*, 2022 ▸; Friedel *et al.*, 1989 ▸), anti­fungal (da Rosa Monte Machado *et al.*, 2020 ▸), anti­protozoal (Clioquinol) (LeVine *et al.*, 2009 ▸), anti­malarial (Chloro­quine) (Orive *et al.*, 2003 ▸) and anti­psychotic (Aripiprazole) (Afzal *et al.*, 2015 ▸; Kaur *et al.*, 2010 ▸; Kumar *et al.*, 2009 ▸; Zajdel *et al.*, 2013 ▸) agents. Halo­quinoline compounds, particularly chloro-substituted ones, are attracting inter­est because the halogen atom is potentially crucial to the bioactivity of the compound and in addition opens up the possibility for further structure elaboration (Majumdar *et al.*, 2011 ▸; Zhang *et al.*, 2010 ▸). Several quinoline-based hybrids linked to other biological moieties *via* hydrazone have been shown to have high biological activity (Katariya *et al.*, 2020 ▸). The class of organic compounds known as hydrazones, which are related to ketones and aldehydes, has the formula *R*_1_ *R*_2_C=NNH_2_ (Kajal *et al.*, 2014 ▸; Marcucci Ribeiro, 2004 ▸). These substances have a variety of biological and pharmacological properties, including anti­microbial, anti-inflammatory, analgesic, anti­fungal, anti­tubercular, anti­viral, anti­cancer (Nasr *et al.*, 2018 ▸), anti­platelet, anti­malarial, anti­convulsant, cardio-protective, anthelmintic, anti­protozoal (Rollas & Küçükgüzel, 2007 ▸), anti­trypanosomal (Narang *et al.*, 2012 ▸) and anti­schistosomiasis activity. The combination of hydrazones with the quinoline nucleus leads to compounds with unique biological and pharmacological activities. In this context, the present investigation reports the synthesis, crystal structures, and IR, ^1^H NMR, ^13^C NMR and mass spectroscopic, and elemental analyses of two diastereoselective derivatives, namely, (*E*)-*N*′-(4-chloro-3-nitro­benzyl­idene)aceto­hy­dra­zide (**IV**) and (*E*)-2-(4-chloro­benzyl­idene)-1-(quin­o­lin-8-yl)hy­dra­zine (**VII**).

## 2. Structural commentary

**Figure 1:**
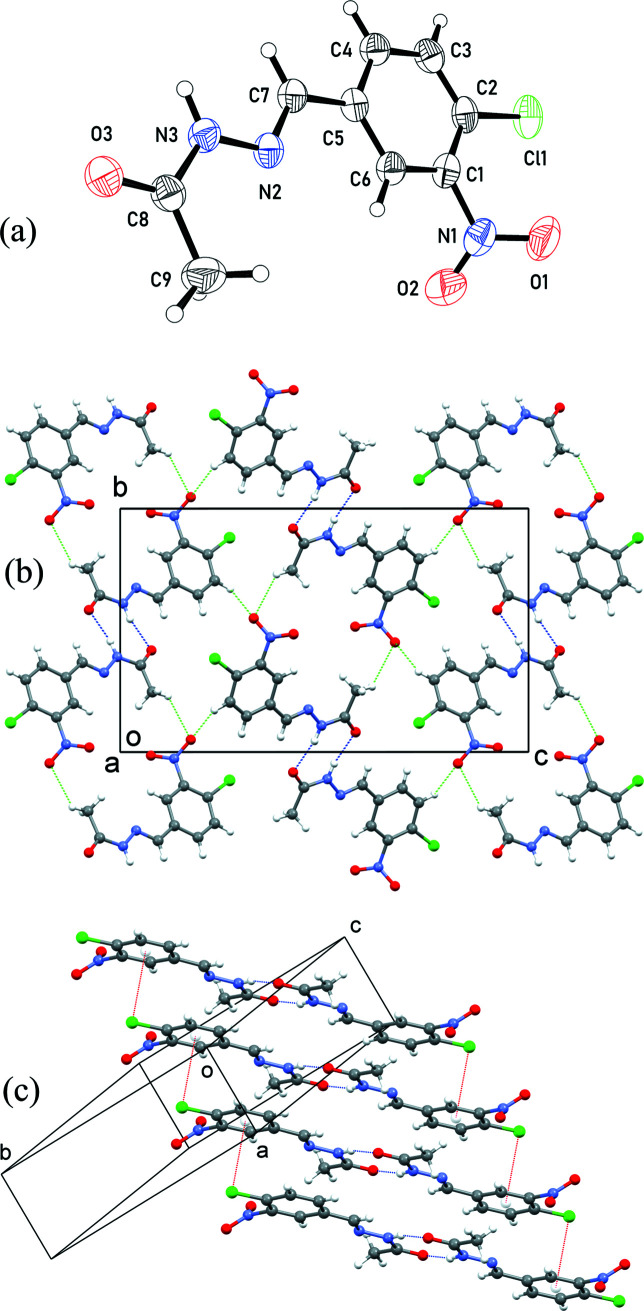
(*a*) The mol­ecular structure of compound **IV**, showing 50% probability displacement ellipsoids for one component of the disordered nitro group. (*b*) The crystal packing viewed down the *a* axis, showing the N—H⋯O hydrogen bonds as blue dotted lines, the C—H⋯O contacts as green dotted lines and the Cl⋯π contacts as red dotted lines. (*c*) A stack of N—H⋯O hydrogen-bonded mol­ecular pairs.

The crystal structure of **IV** is monoclinic, *P*2_1_/*c*. The asymmetric unit of the crystal structure consists of a single mol­ecule [Fig. 1 ▸(*a*)]. Apart from the nitro group and the H atoms of the methyl group, the mol­ecule of **IV** is planar, with a maximum deviation of 0.11 Å for atom Cl1 from the least-squares plane through all the atoms. The nitro group shows positional disorder in the crystal structure (details are available in the *Refinement* section). The nitro group deviates from the plane through the rest of the mol­ecule by a twist around the C1—N1 bond of 49.3 (1)° for the major component and 57.1 (5)° for the minor component. The mol­ecular planarity and twist of the nitro group are consistent with the conformation reported for other structures containing the [(4-chloro-3-nitro­phen­yl)methyl­idene]formohydrazide moiety (Gu *et al.*, 2012 ▸; Mokhnache & Bourzami, 2020 ▸).

**Figure 2:**
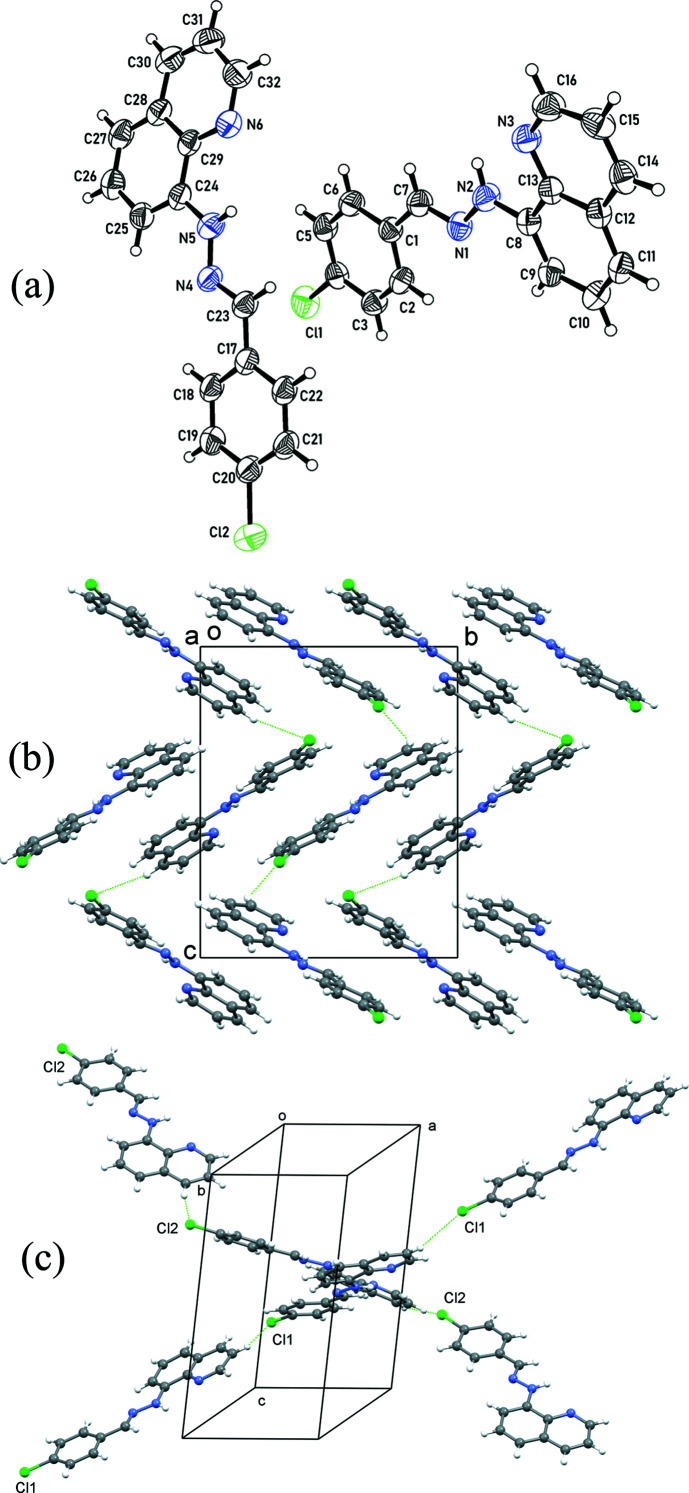
(*a*) The mol­ecular structure of compound **VII**, showing 50% probability displacement ellipsoids. (*b*) The crystal packing, viewed down the *a* axis, showing the C—H⋯Cl contacts as green dotted lines. (*c*) A segment of the crystal structure showing the mol­ecular chains formed through C—H⋯Cl contacts.

The crystal structure of **VII** is monoclinic, *Pn*, and comprises two independent mol­ecules (mol­ecule 1: atoms C1–C16, N1–N3 and Cl1; mol­ecule 2: C17–C32, N4–N6 and Cl2) of the compound [Fig. 2 ▸(*a*)]. The two mol­ecules are planar, with maximum deviations of 0.229 (3) (for N2) and 0.290 (1) Å (for N5) from the least-squares planes through all the atoms of the respective mol­ecules. Intra­molecular N—H⋯N contacts are observed in the structure, with geometry N2—H2*A*⋯N3 = 104.0° and N2⋯N3 = 2.672 (3) Å for the first mol­ecule, and N5—H5*A*⋯N6 = 103.5° and N5⋯N6 = 2.679 (4) Å for the second mol­ecule.

## 3. Supra­molecular features

In the crystal structure of **IV**, neighbouring pairs of mol­ecules, related by inversion symmetry, are linked by two inter­molecular (N3—H3*A*⋯O3) hydrogen bonds [Table 1 ▸ and Fig. 1 ▸(*b*)]. The two hydrogen bonds form rings with 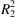 (8) geometry (Etter *et al.*, 1990 ▸; Bernstein *et al.*, 1995 ▸) between the mol­ecules. The linked mol­ecular pairs form columns along the *a* axis of the crystal [Fig. 1 ▸(*c*)] guided by C-halogen⋯π inter­actions (Prasanna & Guru Row, 2000 ▸; Mitra *et al.*, 2020 ▸), with Cl⋯ring-centroid distances of 3.51 Å. Within a stack, the planes of the mol­ecules are parallel and close to either the (12 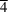 ) or ( 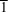 24) plane. C—H⋯O contacts are also observed in the structure, as shown in Table 1 ▸ and Fig. 1 ▸(*b*).

The mol­ecules of compound **VII** are arranged in a herringbone pattern in the crystal [Fig. 2 ▸(*b*)]. Mol­ecules of the same type (*i.e.* mol­ecule 1 or 2) are linked through C—H⋯Cl contacts (Table 2 ▸) to form zigzag chains. The chains are roughly aligned in the direction of [101] and [20 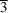 ] [Fig. 2 ▸(*c*)].

## 4. Database survey

De­hydro­abietic acid {systematic name: 2-[(4-chloro-3-nitro­phen­yl)methyl­ene]hydrazide} ethanol solvate [Cambridge Structural Database (CSD; Groom *et al.*, 2016 ▸) refcode VAZYAY; Gu *et al.*, 2012 ▸] and *N*′-[(4-chloro-3-nitro­phen­yl)methyl­idene]pyridine-4-carbohydrazide (ZUTTUG; Mokhnache & Bourzami, 2020 ▸) contain the [(4-chloro-3-nitro­phen­yl)methyl­idene]formohydrazide moiety. Similar to **IV**, the group is planar, except for the *meta*-nitro group, which is twisted from the plane of the rest of the fragment by about 48°.

(*E*)-1-(4-Chloro­benzyl­idene)-2-phenyl­hydrazine (AYUSOD; Tahir *et al.*, 2011 ▸) contains the (1*E*)-1-[(4-chloro­phen­yl)methyl­idene]-2-phenyl­hydrazine group. The planarity of the mol­ecule in **VII** is similar to the geometry observed for the [(4-chloro­phen­yl)methyl­idene]-2-phenyl­hydrazine moiety in GAZYIR (Ojala *et al.*, 2012 ▸) and AYUSOD.

## 5. Experimental details

### 5.1. Compound II: 1-[(2-chloro­quinolin-3-yl)methyl­idene]hydrazine

2-Chloro­quinoline-3-carbaldehyde, **I** (191.61 mg, 0.001 mmol), was dissolved in ethanol (30 ml) and hydrazine hydrate (0.486 ml, 0.01 mmol) was added dropwise. The reaction mixture was refluxed for 3 h followed by solvent evaporation and cooling. The resultant yellow solid was filtered off and washed with a small amount of ethanol before recrystallization from ethanol to afford a yellow powder (see Scheme 1) (Abd-El-Maksoud *et al.*, 2016 ▸). 
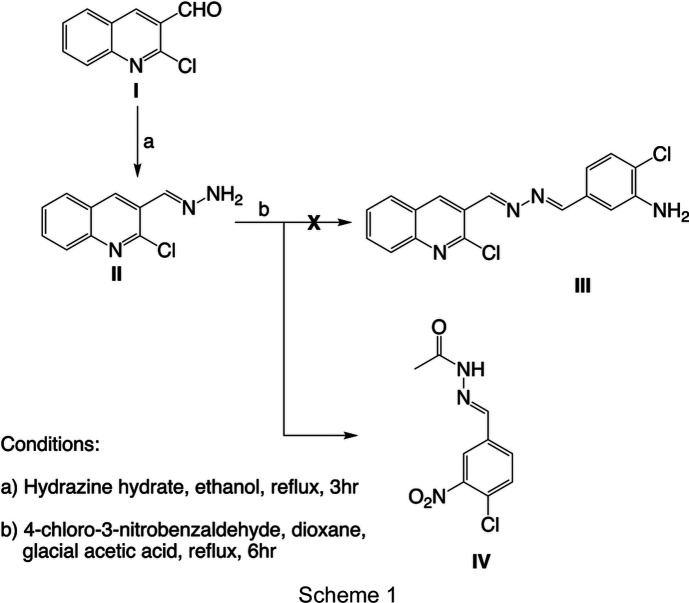


### 5.2. Compound IV: (*E*)-*N*′-(4-chloro-3-nitro­benzyl­idene)acetohydrazide

4-Chloro-3-nitro­benzaldehyde (185.56 mg, 0.001 mmol) and glacial acetic acid (1 ml) were added to a solution of com­pound **II** (205.64 mg, 0.001 mmol) in dioxane (15 ml) while stirring. The reaction mixture was refluxed for 6 h and then cooled and poured into ice water. The solid obtained was crystallized from chloro­form to give yellow crystals of compound **IV** (65% yield) instead of the desired compound **III** (Ibrahim *et al.*, 2010 ▸).

M.p. 245 °C. IR (KBr, ν cm^−1^): 3188 (NH), 3098 (CH aromatic), 2970 (CH aliphatic), 1670 (C=O), 1608 (C=N), 1529, 1352 (NO_2_). ^1^H NMR (DMSO-*d*_6_, 400 MHz): δ 11.50 (*s*, 1H, NH, D_2_O exchangeable), 8.32 (*br*, 1H, H-2′), 8.02 (*s*, 1H, CH=N), 7.97 (*d*, 1H, *J* = 8.4 Hz, H-6′), 7.81 (*d*, 1H, *J* = 8.4 Hz, H-5′), 2.22 (*s*, 3H, CH_3_); MS (EI) *m*/*z* (%): 241, 243 (*M* ^+^, 36.46, 18.06); 59 (C_2_H_5_NO, 100), 43 (C_2_H_3_O, 99.22); analysis calculated (%) for C_9_H_8_ClN_3_O_3_: C 44.74, H 3.34, N 17.39; found; C 44.98, H 3.50, N 17.61.

### 5.3. Compound VI: 1-(quinolin-8-yl)hydrazine

8-Hy­droxy­quinoline, **V** (145.158 mg, 0.001 mmol), was added to hydrazine hydrate (0.486 ml, 0.01 mmol) and the reaction mixture was refluxed for 48 h. The product crystallized as the reaction mixture was slowly cooled to room temperature. The yellow crystalline product isolated by vacuum filtration, followed by washing with warm water and air drying was 8-hydrazino­quinoline **VI** (see Scheme 2) (Guo *et al.*, 2020 ▸; Taylor *et al.*, 2017 ▸).

### 5.4. Compound VII: (*E*)-2-(4-chloro­benzyl­idene)-1-(quinolin-8-yl)hydrazine

4-Chloro­benzaldehyde (140.57 mg, 0.001 mmol) and glacial acetic acid (1 ml) were first added to a solution of 1-(quinolin-8-yl)hydrazine, **VI** (159.18 mg, 0.001 mmol), in ethanol (10 ml). The reaction mixture was refluxed for 8 h, then cooled to room temperature. The solid obtained was filtered off, washed with cold ethanol and recrystallized from ethanol to afford brown crystals (70% yield) of the target compound **VII**. 
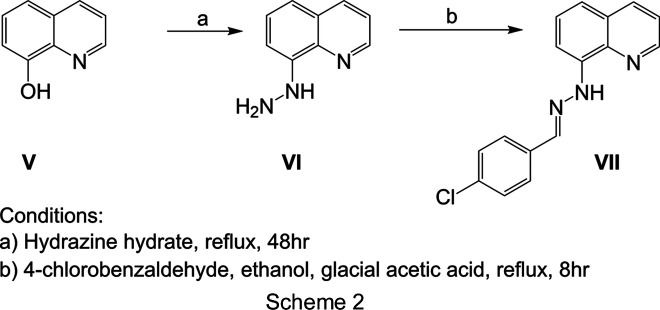


M.p. 126–128 °C; IR (KBr, ν cm^−1^): 3301 (NH), 3037 (CH aromatic), 2942 (CH aliphatic), 1576, 1518 (2C=N); ^1^H NMR (DMSO-*d*_6_, 400 MHz): δ 10.83 (*s*, 1H, NH, D_2_O exchangeable), 8.83 (*d*, 1H, *J* = 2.8 Hz, quinoline-H), 8.39 (*s*, 1H, CH=N), 8.29 (*d*, 1H, *J* = 8 Hz, quinoline-H), 7.72 (*d*, 2H, *J* = 8.4 Hz, Ar-Hs), 7.65 (*d*, 1H, *J* = 7.2 Hz, quinoline-H), 7.56–7.54 (*dd*, 1H, *J* = 8, 4.8 Hz, quinoline-H), 7.52 (*t* like, 1H, *J* = 8.4, 7.6 Hz, quinoline-H), 7.46 (*d*, 2H, *J* = 8.4 Hz, Ar-Hs), 7.33 (*d*, 1H, *J* = 8 Hz, quinoline-H); ^13^C NMR (DMSO-*d*_6_, 100 MHz): δ 14 carbon type, 140.95, 136.60, 136.50, 135.16, 133.05, 129.23, 128.78, 128.11, 127.95, 122.29, 117.20, 108.31, 148.07 (C=N Ar-c), 139.15 (C=N); MS (EI) *m*/*z* (%): 281.76 (*M* ^+^, 50.00), 217 (100, C_11_H_8_ClN_3_); analysis calculated (%) for C_16_H_12_ClN_3_: C 68.21, H 4.29, N 14.91; found: C 68.43, H 4.38; N 15.17.

## 6. Refinement

Crystal and structure refinement data are shown in Table 3 ▸. The nitro group in **IV** is disordered, with the two components related by a 75.0 (6)° twist about the C—N bond, with occupancies of 0.837 (4) and 0.163 (4). H atoms were inserted in idealized positions and a riding model was used, with *U*_iso_(H) values set at 1.2 or 1.5 times the *U*_eq_ value of the atom to which they are bonded.

## Supplementary Material

Crystal structure: contains datablock(s) IV, VII, global. DOI: 10.1107/S2056989023006412/jq2029sup1.cif

Structure factors: contains datablock(s) IV. DOI: 10.1107/S2056989023006412/jq2029IVsup2.hkl

Supporting information file. DOI: 10.1107/S2056989023006412/jq2029IVsup4.cml

Structure factors: contains datablock(s) VII. DOI: 10.1107/S2056989023006412/jq2029VIIsup3.hkl

Supporting information file. DOI: 10.1107/S2056989023006412/jq2029VIIsup5.cml

CCDC references: 2235324, 2235323

Additional supporting information:  crystallographic information; 3D view; checkCIF report

## Figures and Tables

**Table 1:** Hydrogen-bond geometry (Å, °) for **IV** [Chem scheme1]

*D*—H⋯*A*	*D*—H	H⋯*A*	*D*⋯*A*	*D*—H⋯*A*
N3—H3*A*⋯O3^i^	0.86	2.04	2.8803 (18)	167
C3—H3⋯O1^ii^	0.93	2.61	3.442 (3)	149
C9—H9*A*⋯O1*A* ^iii^	0.96	2.30	2.906 (8)	120

Symmetry codes: (i) 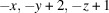 ; (ii) 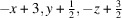 ; (iii) 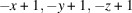 .

**Table 2:** Hydrogen-bond geometry (Å, °) for **VII** [Chem scheme1]

*D*—H⋯*A*	*D*—H	H⋯*A*	*D*⋯*A*	*D*—H⋯*A*
C15—H15⋯Cl1^i^	0.93	3.04	3.779 (3)	138
C30—H30⋯Cl2^ii^	0.93	3.05	3.943 (3)	163

Symmetry codes: (i) 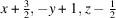 ; (ii) 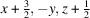 .

**Table 3:** Experimental details For both structures: *Z* = 4. Experiments were carried out with Cu *K*α radiation using a Rigaku OD SuperNova Dual source diffractometer with an Atlas detector. The absorption corrections were Gaussian (*CrysAlis PRO*; Rigaku OD, 2022 ▸). H-atom parameters were constrained.

	**IV**	**VII**
Crystal data		
Chemical formula	C_9_H_8_ClN_3_O_3_	C_16_H_12_ClN_3_
*M*_r_	241.63	281.74
Crystal system, space group	Monoclinic, *P*2_1_/*c*	Monoclinic, *P* *n*
Temperature (K)	293	296
*a*, *b*, *c* (Å)	4.4717 (1), 11.9367 (2), 20.1382 (3)	7.7968 (3), 12.0926 (4), 14.8738 (5)
β (°)	95.689 (2)	100.601 (3)
*V* (Å^3^)	1069.63 (3)	1378.42 (8)
μ (mm^−1^)	3.17	2.38
Crystal size (mm)	0.34 × 0.21 × 0.06	0.26 × 0.14 × 0.07

Data collection		
*T*_min_, *T*_max_	0.500, 1.000	0.713, 1.000
No. of measured, independent and observed [*I* > 2σ(*I*)] reflections	7967, 2107, 1960	11753, 3869, 3609
*R*_int_	0.021	0.024
(sin θ/λ)_max_ (Å^−1^)	0.619	0.619

Refinement		
*R*[*F* ^2^ > 2σ(*F* ^2^)], *wR*(*F* ^2^), *S*	0.038, 0.111, 1.08	0.031, 0.085, 1.04
No. of reflections	2107	3869
No. of parameters	174	361
No. of restraints	132	2
Δρ_max_, Δρ_min_ (e Å^−3^)	0.30, −0.27	0.12, −0.14
Absolute structure	–	Flack *x* determined using 995 quotients [(*I* ^+^) − (*I* ^−^)]/[(*I* ^+^) + (*I* ^−^)] (Parsons *et al.*, 2013 ▸)
Absolute structure parameter	–	0.016 (12)

Computer programs: *CrysAlis PRO* (Rigaku OD, 2022 ▸), *SHELXT* (Sheldrick, 2015*a* ▸), *SHELXL2018* (Sheldrick, 2015*b* ▸), *ORTEP-3 for Windows* (Farrugia, 2012 ▸) and *Mercury* (Macrae *et al.*, 2020 ▸).

## supplementary crystallographic information

### (E)-N'-(4-Chloro-3-nitrobenzylidene)acetohydrazide (IV) Crystal data

**Table:**

C_9_H_8_ClN_3_O_3_	*F*(000) = 496
*M**_r_* = 241.63	*D*_x_ = 1.500 Mg m^−^^3^
Monoclinic, *P*2_1_/*c*	Cu *K*α radiation, λ = 1.54184 Å
*a* = 4.4717 (1) Å	Cell parameters from 5559 reflections
*b* = 11.9367 (2) Å	θ = 4.3–72.9°
*c* = 20.1382 (3) Å	µ = 3.17 mm^−^^1^
β = 95.689 (2)°	*T* = 293 K
*V* = 1069.63 (3) Å^3^	Plate, yellow
*Z* = 4	0.34 × 0.21 × 0.06 mm

### (E)-N'-(4-Chloro-3-nitrobenzylidene)acetohydrazide (IV) Data collection

**Table:**

Rigaku OD SuperNova Dual source diffractometer with an Atlas detector	1960 reflections with *I* > 2σ(*I*)
ω scans	*R*_int_ = 0.021
Absorption correction: gaussian (CrysAlis PRO; Rigaku OD, 2022)	θ_max_ = 72.6°, θ_min_ = 4.3°
*T*_min_ = 0.500, *T*_max_ = 1.000	*h* = −5→4
7967 measured reflections	*k* = −11→14
2107 independent reflections	*l* = −24→23

### (E)-N'-(4-Chloro-3-nitrobenzylidene)acetohydrazide (IV) Refinement

**Table:**

Refinement on *F*^2^	132 restraints
Least-squares matrix: full	Hydrogen site location: inferred from neighbouring sites
*R*[*F*^2^ > 2σ(*F*^2^)] = 0.038	H-atom parameters constrained
*wR*(*F*^2^) = 0.111	*w* = 1/[σ^2^(*F*_o_^2^) + (0.0628*P*)^2^ + 0.2562*P*] where *P* = (*F*_o_^2^ + 2*F*_c_^2^)/3
*S* = 1.08	(Δ/σ)_max_ = 0.001
2107 reflections	Δρ_max_ = 0.30 e Å^−^^3^
174 parameters	Δρ_min_ = −0.26 e Å^−^^3^

### (E)-N'-(4-Chloro-3-nitrobenzylidene)acetohydrazide (IV) Special details

**Table:**

Geometry. All esds (except the esd in the dihedral angle between two l.s. planes) are estimated using the full covariance matrix. The cell esds are taken into account individually in the estimation of esds in distances, angles and torsion angles; correlations between esds in cell parameters are only used when they are defined by crystal symmetry. An approximate (isotropic) treatment of cell esds is used for estimating esds involving l.s. planes.
Refinement. Single-crystal XRD data were collected on an Agilent SuperNova Dual Atlas diffractometer with a mirror monochromator using Cu radiation. Crystal structures were solved and refined using *SHELXT* (Sheldrick, 2015a) and *SHELXL* (Sheldrick, 2015b). Non-hydrogen atoms for both IV and VII were refined with anisotropic displacement parameters.

### (E)-N'-(4-Chloro-3-nitrobenzylidene)acetohydrazide (IV) Fractional atomic coordinates and isotropic or equivalent isotropic displacement parameters (Å^2^)

**Table:**

	*x*	*y*	*z*	*U*_iso_*/*U*_eq_	Occ. (<1)
C1	1.1687 (3)	0.64078 (14)	0.65566 (9)	0.0471 (4)	0.837 (4)
N1	1.2480 (5)	0.52916 (17)	0.63231 (17)	0.0625 (7)	0.837 (4)
O1	1.2534 (7)	0.45143 (16)	0.67293 (12)	0.0952 (8)	0.837 (4)
O2	1.2958 (7)	0.51630 (18)	0.57543 (12)	0.1103 (10)	0.837 (4)
C1A	1.1687 (3)	0.64078 (14)	0.65566 (9)	0.0471 (4)	0.163 (4)
N1A	1.2420 (17)	0.5222 (3)	0.6464 (8)	0.068 (3)	0.163 (4)
O1A	1.034 (2)	0.4543 (3)	0.6471 (9)	0.116 (4)	0.163 (4)
O2A	1.4995 (17)	0.4946 (6)	0.6475 (7)	0.090 (3)	0.163 (4)
C2	1.3004 (3)	0.68734 (15)	0.71455 (8)	0.0482 (4)	
C3	1.2216 (4)	0.79476 (16)	0.73109 (8)	0.0512 (4)	
H3	1.313563	0.828395	0.769479	0.061*	
C4	1.0057 (4)	0.85255 (15)	0.69064 (8)	0.0472 (4)	
H4	0.952715	0.924666	0.702441	0.057*	
C5	0.8667 (3)	0.80468 (13)	0.63268 (7)	0.0411 (3)	
C6	0.9539 (3)	0.69802 (13)	0.61454 (8)	0.0450 (4)	
H6	0.868744	0.665462	0.575164	0.054*	
C7	0.6293 (3)	0.86655 (13)	0.59289 (8)	0.0415 (3)	
H7	0.572080	0.936881	0.606968	0.050*	
C8	0.1213 (3)	0.85608 (14)	0.44985 (8)	0.0446 (3)	
C9	0.2010 (5)	0.74632 (18)	0.42125 (11)	0.0691 (5)	
H9A	0.062133	0.729457	0.383109	0.104*	
H9B	0.191210	0.688737	0.454244	0.104*	
H9C	0.401094	0.749991	0.407961	0.104*	
N2	0.5005 (3)	0.82510 (11)	0.53927 (6)	0.0419 (3)	
N3	0.2774 (3)	0.88996 (11)	0.50721 (7)	0.0428 (3)	
H3A	0.235971	0.953659	0.524035	0.051*	
O3	−0.0780 (3)	0.91708 (11)	0.42345 (6)	0.0548 (3)	
Cl1	1.56369 (10)	0.61768 (4)	0.76803 (3)	0.0685 (2)	

### (E)-N'-(4-Chloro-3-nitrobenzylidene)acetohydrazide (IV) Atomic displacement parameters (Å^2^)

**Table:**

	*U*^11^	*U*^22^	*U*^33^	*U*^12^	*U*^13^	*U*^23^
C1	0.0399 (7)	0.0448 (8)	0.0565 (9)	−0.0002 (6)	0.0039 (7)	0.0106 (7)
N1	0.0628 (13)	0.0481 (11)	0.0749 (17)	0.0061 (10)	−0.0019 (10)	0.0087 (10)
O1	0.137 (2)	0.0523 (11)	0.0910 (15)	0.0084 (12)	−0.0170 (14)	0.0188 (10)
O2	0.171 (2)	0.0700 (13)	0.0966 (17)	0.0287 (14)	0.0491 (17)	−0.0050 (11)
C1A	0.0399 (7)	0.0448 (8)	0.0565 (9)	−0.0002 (6)	0.0039 (7)	0.0106 (7)
N1A	0.071 (5)	0.055 (5)	0.078 (5)	0.002 (4)	0.007 (4)	0.015 (4)
O1A	0.122 (8)	0.077 (6)	0.146 (8)	−0.015 (6)	0.003 (7)	−0.005 (6)
O2A	0.072 (5)	0.060 (5)	0.138 (7)	0.027 (4)	0.010 (5)	−0.007 (5)
C2	0.0362 (7)	0.0569 (9)	0.0509 (9)	−0.0012 (6)	0.0014 (6)	0.0177 (7)
C3	0.0457 (8)	0.0625 (10)	0.0435 (8)	−0.0052 (7)	−0.0046 (7)	0.0048 (7)
C4	0.0446 (8)	0.0496 (9)	0.0464 (8)	−0.0002 (7)	0.0003 (6)	0.0018 (7)
C5	0.0343 (7)	0.0465 (8)	0.0421 (7)	−0.0018 (6)	0.0021 (6)	0.0084 (6)
C6	0.0418 (7)	0.0462 (8)	0.0461 (8)	−0.0028 (6)	−0.0005 (6)	0.0049 (6)
C7	0.0356 (7)	0.0437 (8)	0.0447 (8)	0.0003 (6)	0.0016 (6)	0.0053 (6)
C8	0.0418 (7)	0.0490 (8)	0.0422 (8)	−0.0022 (6)	−0.0003 (6)	0.0015 (6)
C9	0.0786 (13)	0.0622 (11)	0.0628 (11)	0.0109 (10)	−0.0116 (10)	−0.0160 (9)
N2	0.0359 (6)	0.0448 (7)	0.0444 (7)	0.0028 (5)	0.0004 (5)	0.0067 (5)
N3	0.0396 (6)	0.0429 (7)	0.0443 (7)	0.0049 (5)	−0.0044 (5)	0.0006 (5)
O3	0.0522 (6)	0.0591 (7)	0.0494 (6)	0.0066 (5)	−0.0138 (5)	−0.0016 (5)
Cl1	0.0502 (3)	0.0778 (4)	0.0735 (3)	0.0036 (2)	−0.0134 (2)	0.0291 (2)

### (E)-N'-(4-Chloro-3-nitrobenzylidene)acetohydrazide (IV) Geometric parameters (Å, º)

**Table:**

C1—C6	1.385 (2)	C4—H4	0.9300
C1—C2	1.387 (2)	C5—C6	1.391 (2)
C1—N1	1.468 (3)	C5—C7	1.465 (2)
N1—O2	1.196 (4)	C6—H6	0.9300
N1—O1	1.236 (3)	C7—N2	1.273 (2)
C1A—C6	1.385 (2)	C7—H7	0.9300
C1A—C2	1.387 (2)	C8—O3	1.230 (2)
C1A—N1A	1.469 (3)	C8—N3	1.351 (2)
N1A—O2A	1.196 (4)	C8—C9	1.489 (3)
N1A—O1A	1.236 (4)	C9—H9A	0.9600
C2—C3	1.379 (3)	C9—H9B	0.9600
C2—Cl1	1.7281 (15)	C9—H9C	0.9600
C3—C4	1.384 (2)	N2—N3	1.3726 (17)
C3—H3	0.9300	N3—H3A	0.8600
C4—C5	1.390 (2)		
			
C6—C1—C2	121.62 (15)	C4—C5—C6	118.90 (14)
C6—C1—N1	115.56 (19)	C4—C5—C7	119.45 (14)
C2—C1—N1	122.81 (18)	C6—C5—C7	121.64 (14)
O2—N1—O1	123.0 (2)	C1A—C6—C5	119.37 (15)
O2—N1—C1	119.7 (2)	C1—C6—C5	119.37 (15)
O1—N1—C1	117.3 (3)	C1—C6—H6	120.3
C6—C1A—C2	121.62 (15)	C5—C6—H6	120.3
C6—C1A—N1A	123.4 (6)	N2—C7—C5	120.71 (14)
C2—C1A—N1A	114.4 (6)	N2—C7—H7	119.6
O2A—N1A—O1A	122.9 (3)	C5—C7—H7	119.6
O2A—N1A—C1A	119.4 (3)	O3—C8—N3	118.88 (15)
O1A—N1A—C1A	117.0 (3)	O3—C8—C9	123.02 (15)
C3—C2—C1	118.81 (14)	N3—C8—C9	118.10 (15)
C3—C2—C1A	118.81 (14)	C8—C9—H9A	109.5
C3—C2—Cl1	118.12 (13)	C8—C9—H9B	109.5
C1—C2—Cl1	123.06 (14)	H9A—C9—H9B	109.5
C1A—C2—Cl1	123.06 (14)	C8—C9—H9C	109.5
C2—C3—C4	120.07 (16)	H9A—C9—H9C	109.5
C2—C3—H3	120.0	H9B—C9—H9C	109.5
C4—C3—H3	120.0	C7—N2—N3	115.24 (13)
C3—C4—C5	121.15 (16)	C8—N3—N2	121.54 (13)
C3—C4—H4	119.4	C8—N3—H3A	119.2
C5—C4—H4	119.4	N2—N3—H3A	119.2

### (E)-N'-(4-Chloro-3-nitrobenzylidene)acetohydrazide (IV) Hydrogen-bond geometry (Å, º)

**Table:**

*D*—H···*A*	*D*—H	H···*A*	*D*···*A*	*D*—H···*A*
N3—H3*A*···O3^i^	0.86	2.04	2.8803 (18)	167
C3—H3···O1^ii^	0.93	2.61	3.442 (3)	149
C9—H9*A*···O1*A*^iii^	0.96	2.30	2.906 (8)	120

Symmetry codes: (i) −*x*, −*y*+2, −*z*+1; (ii) −*x*+3, *y*+1/2, −*z*+3/2; (iii) −*x*+1, −*y*+1, −*z*+1.

### (E)-2-(4-Chlorobenzylidene)-1-(quinolin-8-yl)hydrazine (VII) Crystal data

**Table:**

C_16_H_12_ClN_3_	*F*(000) = 584
*M**_r_* = 281.74	*D*_x_ = 1.358 Mg m^−^^3^
Monoclinic, *P**n*	Cu *K*α radiation, λ = 1.54184 Å
*a* = 7.7968 (3) Å	Cell parameters from 6539 reflections
*b* = 12.0926 (4) Å	θ = 3.6–72.7°
*c* = 14.8738 (5) Å	µ = 2.38 mm^−^^1^
β = 100.601 (3)°	*T* = 296 K
*V* = 1378.42 (8) Å^3^	Plate, brown
*Z* = 4	0.26 × 0.14 × 0.07 mm

### (E)-2-(4-Chlorobenzylidene)-1-(quinolin-8-yl)hydrazine (VII) Data collection

**Table:**

Rigaku OD SuperNova Dual source diffractometer with an Atlas detector	3609 reflections with *I* > 2σ(*I*)
ω scans	*R*_int_ = 0.024
Absorption correction: gaussian (CrysAlis PRO; Rigaku OD, 2022)	θ_max_ = 72.8°, θ_min_ = 3.7°
*T*_min_ = 0.713, *T*_max_ = 1.000	*h* = −9→6
11753 measured reflections	*k* = −14→14
3869 independent reflections	*l* = −18→18

### (E)-2-(4-Chlorobenzylidene)-1-(quinolin-8-yl)hydrazine (VII) Refinement

**Table:**

Refinement on *F*^2^	Hydrogen site location: inferred from neighbouring sites
Least-squares matrix: full	H-atom parameters constrained
*R*[*F*^2^ > 2σ(*F*^2^)] = 0.031	*w* = 1/[σ^2^(*F*_o_^2^) + (0.0484*P*)^2^ + 0.0525*P*] where *P* = (*F*_o_^2^ + 2*F*_c_^2^)/3
*wR*(*F*^2^) = 0.085	(Δ/σ)_max_ < 0.001
*S* = 1.04	Δρ_max_ = 0.12 e Å^−^^3^
3869 reflections	Δρ_min_ = −0.14 e Å^−^^3^
361 parameters	Absolute structure: Flack *x* determined using 995 quotients [(I+)-(I-)]/[(I+)+(I-)] (Parsons *et al.*, 2013)
2 restraints	Absolute structure parameter: 0.016 (12)

### (E)-2-(4-Chlorobenzylidene)-1-(quinolin-8-yl)hydrazine (VII) Special details

**Table:**

Geometry. All esds (except the esd in the dihedral angle between two l.s. planes) are estimated using the full covariance matrix. The cell esds are taken into account individually in the estimation of esds in distances, angles and torsion angles; correlations between esds in cell parameters are only used when they are defined by crystal symmetry. An approximate (isotropic) treatment of cell esds is used for estimating esds involving l.s. planes.
Refinement. Single-crystal XRD data were collected on an Agilent SuperNova Dual Atlas diffractometer with a mirror monochromator using Cu radiation. Crystal structures were solved and refined using *SHELXT* (Sheldrick, 2015a) and *SHELXL* (Sheldrick, 2015b). Non-hydrogen atoms for both IV and VII were refined with anisotropic displacement parameters.

### (E)-2-(4-Chlorobenzylidene)-1-(quinolin-8-yl)hydrazine (VII) Fractional atomic coordinates and isotropic or equivalent isotropic displacement parameters (Å^2^)

**Table:**

	*x*	*y*	*z*	*U*_iso_*/*U*_eq_	
C1	0.6750 (3)	0.4572 (2)	0.58792 (17)	0.0527 (5)	
C2	0.5178 (4)	0.5151 (2)	0.58122 (18)	0.0576 (6)	
H2	0.507242	0.585152	0.554930	0.069*	
C3	0.3785 (4)	0.4695 (2)	0.61317 (19)	0.0591 (6)	
H3	0.274364	0.508481	0.608568	0.071*	
C4	0.3949 (4)	0.3653 (2)	0.65215 (17)	0.0559 (6)	
C5	0.5470 (4)	0.3067 (2)	0.66004 (18)	0.0618 (6)	
H5	0.556356	0.236933	0.686762	0.074*	
C6	0.6876 (4)	0.3525 (2)	0.62774 (19)	0.0602 (6)	
H6	0.791158	0.312863	0.632762	0.072*	
C7	0.8253 (4)	0.5031 (2)	0.55413 (19)	0.0581 (6)	
H7	0.925239	0.460403	0.556268	0.070*	
C8	0.9657 (3)	0.73712 (19)	0.44787 (16)	0.0506 (5)	
C9	0.8354 (4)	0.8142 (2)	0.44385 (18)	0.0565 (6)	
H9	0.740133	0.800018	0.471623	0.068*	
C10	0.8462 (4)	0.9148 (2)	0.39764 (19)	0.0620 (6)	
H10	0.755828	0.965694	0.394070	0.074*	
C11	0.9862 (4)	0.9395 (2)	0.35786 (19)	0.0592 (6)	
H11	0.990642	1.006567	0.327755	0.071*	
C12	1.1237 (3)	0.8630 (2)	0.36266 (16)	0.0516 (5)	
C13	1.1132 (3)	0.75964 (19)	0.40668 (16)	0.0502 (5)	
C14	1.2766 (4)	0.8827 (2)	0.32737 (18)	0.0604 (6)	
H14	1.292238	0.950267	0.300041	0.072*	
C15	1.4009 (4)	0.8035 (3)	0.3332 (2)	0.0662 (7)	
H15	1.500980	0.815666	0.308877	0.079*	
C16	1.3775 (4)	0.7031 (3)	0.3761 (2)	0.0671 (7)	
H16	1.463862	0.649448	0.379133	0.080*	
C17	0.1294 (4)	0.2803 (2)	0.41911 (17)	0.0567 (6)	
C18	−0.0230 (4)	0.2228 (2)	0.4243 (2)	0.0644 (7)	
H18	−0.015814	0.154010	0.452680	0.077*	
C19	−0.1848 (4)	0.2662 (2)	0.3881 (2)	0.0681 (7)	
H19	−0.285878	0.226660	0.391183	0.082*	
C20	−0.1947 (4)	0.3694 (2)	0.34706 (19)	0.0663 (7)	
C21	−0.0454 (4)	0.4295 (2)	0.34266 (19)	0.0661 (7)	
H21	−0.053490	0.499214	0.315895	0.079*	
C22	0.1157 (4)	0.3847 (2)	0.37846 (19)	0.0626 (6)	
H22	0.216388	0.424649	0.375408	0.075*	
C23	0.3017 (4)	0.2337 (2)	0.45288 (18)	0.0607 (6)	
H23	0.400830	0.275359	0.450152	0.073*	
C24	0.5092 (4)	0.0001 (2)	0.56549 (17)	0.0564 (6)	
C25	0.3786 (4)	−0.0730 (2)	0.57278 (19)	0.0637 (7)	
H25	0.264350	−0.057283	0.545057	0.076*	
C26	0.4168 (5)	−0.1719 (2)	0.6221 (2)	0.0712 (8)	
H26	0.327195	−0.221627	0.625588	0.085*	
C27	0.5824 (5)	−0.1965 (2)	0.6649 (2)	0.0715 (8)	
H27	0.604432	−0.261624	0.698298	0.086*	
C28	0.7202 (4)	−0.1232 (2)	0.65860 (17)	0.0605 (6)	
C29	0.6849 (4)	−0.0234 (2)	0.60844 (17)	0.0552 (5)	
C30	0.8961 (5)	−0.1427 (3)	0.6997 (2)	0.0747 (8)	
H30	0.926770	−0.207020	0.733023	0.090*	
C31	1.0200 (5)	−0.0667 (3)	0.6902 (2)	0.0787 (9)	
H31	1.135851	−0.078429	0.717383	0.094*	
C32	0.9717 (4)	0.0294 (3)	0.6392 (2)	0.0743 (8)	
H32	1.058474	0.080213	0.633341	0.089*	
N1	0.8201 (3)	0.60135 (18)	0.52172 (15)	0.0572 (5)	
N2	0.9658 (3)	0.63652 (19)	0.49216 (18)	0.0624 (5)	
H2A	1.058243	0.596165	0.501010	0.075*	
N3	1.2389 (3)	0.68040 (19)	0.41259 (17)	0.0605 (5)	
N4	0.3177 (3)	0.13565 (18)	0.48631 (16)	0.0623 (6)	
N5	0.4832 (3)	0.0983 (2)	0.51623 (18)	0.0675 (6)	
H5A	0.571013	0.135167	0.504734	0.081*	
N6	0.8104 (3)	0.05199 (19)	0.59898 (17)	0.0640 (5)	
Cl1	0.21942 (11)	0.30746 (7)	0.69220 (7)	0.0832 (2)	
Cl2	−0.39863 (14)	0.42416 (8)	0.30136 (9)	0.0979 (3)	

### (E)-2-(4-Chlorobenzylidene)-1-(quinolin-8-yl)hydrazine (VII) Atomic displacement parameters (Å^2^)

**Table:**

	*U*^11^	*U*^22^	*U*^33^	*U*^12^	*U*^13^	*U*^23^
C1	0.0560 (13)	0.0488 (12)	0.0530 (12)	−0.0026 (10)	0.0094 (10)	0.0045 (9)
C2	0.0686 (15)	0.0453 (12)	0.0600 (14)	0.0001 (11)	0.0143 (11)	0.0092 (9)
C3	0.0610 (15)	0.0567 (14)	0.0608 (14)	0.0026 (11)	0.0143 (11)	0.0066 (11)
C4	0.0639 (15)	0.0548 (13)	0.0501 (12)	−0.0117 (11)	0.0136 (11)	0.0029 (9)
C5	0.0776 (18)	0.0491 (13)	0.0581 (14)	−0.0073 (12)	0.0105 (12)	0.0136 (10)
C6	0.0625 (15)	0.0524 (13)	0.0648 (15)	0.0005 (11)	0.0090 (12)	0.0129 (11)
C7	0.0596 (15)	0.0524 (13)	0.0632 (14)	−0.0019 (11)	0.0135 (11)	0.0082 (10)
C8	0.0555 (14)	0.0462 (12)	0.0503 (12)	−0.0053 (10)	0.0101 (10)	−0.0006 (9)
C9	0.0605 (14)	0.0515 (13)	0.0610 (14)	−0.0019 (11)	0.0202 (11)	−0.0030 (10)
C10	0.0706 (17)	0.0487 (13)	0.0676 (15)	0.0084 (11)	0.0155 (13)	−0.0005 (10)
C11	0.0727 (16)	0.0430 (12)	0.0621 (14)	−0.0048 (11)	0.0127 (12)	0.0028 (10)
C12	0.0610 (14)	0.0474 (12)	0.0456 (11)	−0.0083 (10)	0.0078 (9)	−0.0011 (9)
C13	0.0546 (13)	0.0469 (12)	0.0484 (12)	−0.0051 (9)	0.0077 (10)	−0.0007 (9)
C14	0.0684 (15)	0.0605 (14)	0.0527 (13)	−0.0159 (13)	0.0124 (11)	0.0020 (10)
C15	0.0547 (15)	0.0821 (19)	0.0638 (15)	−0.0067 (13)	0.0158 (12)	0.0069 (13)
C16	0.0567 (15)	0.0708 (17)	0.0758 (17)	0.0045 (13)	0.0179 (13)	0.0049 (13)
C17	0.0749 (17)	0.0454 (12)	0.0481 (12)	0.0036 (11)	0.0067 (11)	−0.0006 (9)
C18	0.0829 (19)	0.0427 (13)	0.0662 (15)	0.0005 (12)	0.0102 (13)	0.0057 (10)
C19	0.0748 (18)	0.0543 (15)	0.0740 (17)	−0.0041 (13)	0.0106 (13)	0.0027 (12)
C20	0.079 (2)	0.0544 (15)	0.0620 (15)	0.0071 (13)	0.0031 (13)	0.0003 (10)
C21	0.087 (2)	0.0464 (13)	0.0612 (15)	0.0036 (12)	0.0036 (14)	0.0082 (10)
C22	0.0792 (18)	0.0482 (13)	0.0591 (15)	−0.0013 (12)	0.0093 (13)	0.0046 (10)
C23	0.0736 (18)	0.0521 (14)	0.0551 (14)	0.0056 (12)	0.0080 (12)	0.0030 (10)
C24	0.0741 (16)	0.0428 (11)	0.0517 (13)	0.0056 (11)	0.0100 (11)	−0.0026 (9)
C25	0.0721 (18)	0.0552 (14)	0.0617 (16)	0.0007 (12)	0.0068 (12)	−0.0060 (11)
C26	0.089 (2)	0.0533 (15)	0.0721 (17)	−0.0082 (14)	0.0158 (15)	−0.0006 (12)
C27	0.101 (2)	0.0493 (14)	0.0635 (16)	0.0068 (14)	0.0133 (15)	0.0066 (11)
C28	0.0787 (18)	0.0517 (14)	0.0512 (13)	0.0118 (12)	0.0123 (12)	−0.0007 (9)
C29	0.0689 (15)	0.0465 (12)	0.0510 (12)	0.0071 (11)	0.0135 (11)	−0.0054 (9)
C30	0.089 (2)	0.0681 (18)	0.0650 (17)	0.0239 (17)	0.0098 (15)	0.0024 (13)
C31	0.0683 (19)	0.089 (2)	0.0777 (19)	0.0205 (16)	0.0097 (14)	−0.0059 (15)
C32	0.0682 (18)	0.0788 (19)	0.0770 (18)	0.0022 (15)	0.0164 (15)	−0.0103 (14)
N1	0.0635 (13)	0.0498 (11)	0.0611 (12)	−0.0068 (9)	0.0185 (10)	0.0047 (9)
N2	0.0595 (13)	0.0521 (11)	0.0799 (14)	0.0001 (10)	0.0241 (11)	0.0127 (10)
N3	0.0563 (12)	0.0565 (12)	0.0700 (13)	0.0016 (9)	0.0152 (10)	0.0064 (9)
N4	0.0725 (15)	0.0489 (11)	0.0620 (12)	0.0083 (10)	0.0031 (10)	0.0005 (9)
N5	0.0710 (15)	0.0531 (12)	0.0762 (15)	0.0058 (11)	0.0075 (12)	0.0118 (10)
N6	0.0693 (14)	0.0572 (12)	0.0667 (13)	0.0040 (10)	0.0154 (11)	−0.0046 (10)
Cl1	0.0837 (5)	0.0792 (5)	0.0940 (5)	−0.0197 (4)	0.0358 (4)	0.0109 (4)
Cl2	0.0834 (6)	0.0768 (5)	0.1236 (7)	0.0133 (4)	−0.0063 (5)	0.0136 (5)

### (E)-2-(4-Chlorobenzylidene)-1-(quinolin-8-yl)hydrazine (VII) Geometric parameters (Å, º)

**Table:**

C1—C6	1.393 (4)	C17—C23	1.458 (4)
C1—C2	1.399 (4)	C18—C19	1.381 (5)
C1—C7	1.467 (4)	C18—H18	0.9300
C2—C3	1.379 (4)	C19—C20	1.384 (4)
C2—H2	0.9300	C19—H19	0.9300
C3—C4	1.384 (4)	C20—C21	1.384 (5)
C3—H3	0.9300	C20—Cl2	1.741 (3)
C4—C5	1.368 (4)	C21—C22	1.382 (4)
C4—Cl1	1.736 (3)	C21—H21	0.9300
C5—C6	1.390 (4)	C22—H22	0.9300
C5—H5	0.9300	C23—N4	1.283 (4)
C6—H6	0.9300	C23—H23	0.9300
C7—N1	1.281 (3)	C24—C25	1.367 (4)
C7—H7	0.9300	C24—N5	1.391 (3)
C8—C9	1.372 (4)	C24—C29	1.429 (4)
C8—N2	1.383 (3)	C25—C26	1.406 (4)
C8—C13	1.425 (3)	C25—H25	0.9300
C9—C10	1.407 (4)	C26—C27	1.363 (5)
C9—H9	0.9300	C26—H26	0.9300
C10—C11	1.367 (4)	C27—C28	1.409 (5)
C10—H10	0.9300	C27—H27	0.9300
C11—C12	1.408 (4)	C28—C30	1.415 (5)
C11—H11	0.9300	C28—C29	1.419 (4)
C12—C14	1.408 (4)	C29—N6	1.363 (4)
C12—C13	1.421 (3)	C30—C31	1.359 (5)
C13—N3	1.362 (3)	C30—H30	0.9300
C14—C15	1.355 (4)	C31—C32	1.402 (5)
C14—H14	0.9300	C31—H31	0.9300
C15—C16	1.399 (4)	C32—N6	1.318 (4)
C15—H15	0.9300	C32—H32	0.9300
C16—N3	1.325 (4)	N1—N2	1.360 (3)
C16—H16	0.9300	N2—H2A	0.8600
C17—C18	1.391 (4)	N4—N5	1.363 (3)
C17—C22	1.395 (4)	N5—H5A	0.8600
			
C6—C1—C2	118.6 (2)	C17—C18—H18	119.4
C6—C1—C7	119.4 (2)	C18—C19—C20	119.1 (3)
C2—C1—C7	122.1 (2)	C18—C19—H19	120.4
C3—C2—C1	120.7 (2)	C20—C19—H19	120.4
C3—C2—H2	119.7	C21—C20—C19	121.0 (3)
C1—C2—H2	119.7	C21—C20—Cl2	119.8 (2)
C2—C3—C4	119.4 (3)	C19—C20—Cl2	119.2 (3)
C2—C3—H3	120.3	C22—C21—C20	119.3 (2)
C4—C3—H3	120.3	C22—C21—H21	120.4
C5—C4—C3	121.3 (2)	C20—C21—H21	120.4
C5—C4—Cl1	119.20 (19)	C21—C22—C17	120.9 (3)
C3—C4—Cl1	119.5 (2)	C21—C22—H22	119.6
C4—C5—C6	119.4 (2)	C17—C22—H22	119.6
C4—C5—H5	120.3	N4—C23—C17	120.6 (3)
C6—C5—H5	120.3	N4—C23—H23	119.7
C5—C6—C1	120.7 (3)	C17—C23—H23	119.7
C5—C6—H6	119.7	C25—C24—N5	123.7 (3)
C1—C6—H6	119.7	C25—C24—C29	120.2 (2)
N1—C7—C1	120.5 (3)	N5—C24—C29	116.1 (2)
N1—C7—H7	119.7	C24—C25—C26	120.1 (3)
C1—C7—H7	119.7	C24—C25—H25	119.9
C9—C8—N2	123.7 (2)	C26—C25—H25	119.9
C9—C8—C13	120.1 (2)	C27—C26—C25	121.4 (3)
N2—C8—C13	116.3 (2)	C27—C26—H26	119.3
C8—C9—C10	119.9 (2)	C25—C26—H26	119.3
C8—C9—H9	120.1	C26—C27—C28	120.0 (3)
C10—C9—H9	120.1	C26—C27—H27	120.0
C11—C10—C9	121.7 (3)	C28—C27—H27	120.0
C11—C10—H10	119.2	C27—C28—C30	123.9 (3)
C9—C10—H10	119.2	C27—C28—C29	119.6 (3)
C10—C11—C12	119.7 (2)	C30—C28—C29	116.6 (3)
C10—C11—H11	120.1	N6—C29—C28	123.3 (3)
C12—C11—H11	120.1	N6—C29—C24	117.9 (2)
C11—C12—C14	124.0 (2)	C28—C29—C24	118.7 (3)
C11—C12—C13	119.6 (2)	C31—C30—C28	119.6 (3)
C14—C12—C13	116.5 (2)	C31—C30—H30	120.2
N3—C13—C12	123.2 (2)	C28—C30—H30	120.2
N3—C13—C8	117.8 (2)	C30—C31—C32	119.4 (3)
C12—C13—C8	119.1 (2)	C30—C31—H31	120.3
C15—C14—C12	120.1 (2)	C32—C31—H31	120.3
C15—C14—H14	120.0	N6—C32—C31	123.8 (3)
C12—C14—H14	120.0	N6—C32—H32	118.1
C14—C15—C16	119.4 (3)	C31—C32—H32	118.1
C14—C15—H15	120.3	C7—N1—N2	116.2 (2)
C16—C15—H15	120.3	N1—N2—C8	120.1 (2)
N3—C16—C15	123.5 (3)	N1—N2—H2A	120.0
N3—C16—H16	118.3	C8—N2—H2A	120.0
C15—C16—H16	118.3	C16—N3—C13	117.4 (2)
C18—C17—C22	118.6 (3)	C23—N4—N5	116.9 (3)
C18—C17—C23	122.0 (2)	N4—N5—C24	119.5 (2)
C22—C17—C23	119.5 (3)	N4—N5—H5A	120.2
C19—C18—C17	121.1 (2)	C24—N5—H5A	120.2
C19—C18—H18	119.4	C32—N6—C29	117.2 (3)

### (E)-2-(4-Chlorobenzylidene)-1-(quinolin-8-yl)hydrazine (VII) Hydrogen-bond geometry (Å, º)

**Table:**

*D*—H···*A*	*D*—H	H···*A*	*D*···*A*	*D*—H···*A*
C15—H15···Cl1^i^	0.93	3.04	3.779 (3)	138
C30—H30···Cl2^ii^	0.93	3.05	3.943 (3)	163

Symmetry codes: (i) *x*+3/2, −*y*+1, *z*−1/2; (ii) *x*+3/2, −*y*, *z*+1/2.
